# Neutropenia in Patients With Castration‐Resistant Prostate Cancer Treated Using Cabazitaxel and Prophylactic Administration of Pegfilgrastim

**DOI:** 10.1155/aiu/8111761

**Published:** 2026-09-12

**Authors:** Kazuhiko Oshinomi, Shota Kikuchi, Masahiro Kurokawa, Toshiki Mugita, Tatsuki Inoue, Motoki Yamagishi, Yoshihiro Nakagami, Masakazu Nagata, Haruaki Sasaki, Kohzou Fuji, Masashi Morita, Takashi Fukagai

**Affiliations:** ^1^ Department of Urology, School of Medicine, Showa Medical University, Tokyo, Japan; ^2^ Department of Urology, Fujigaoka Hospital, Showa Medical University, Yokohama, Japan; ^3^ Department of Urology, Northern Yokohama Hospital, Showa Medical University, Yokohama, Japan; ^4^ Department of Urology, Koto Toyosu Hospital, Showa Medical University, Tokyo, Japan

**Keywords:** docetaxel, febrile neutropenia, granulocyte colony–stimulating factor, neutropenia, prostate cancer

## Abstract

**Background and Aims:**

Cabazitaxel has demonstrated survival benefits in patients with metastatic castration‐resistant prostate cancer (CRPC). However, Japanese patients are reported to experience higher rates of hematologic toxicity compared to their Caucasian counterparts. Although primary prophylaxis with pegfilgrastim is now routinely used in clinical practice, febrile neutropenia (FN) may still occur. In this study, we aimed to evaluate the real‐world safety of cabazitaxel with universal pegfilgrastim prophylaxis in Japanese patients with CRPC, particularly focusing on FN during the first treatment cycle.

**Methods:**

We retrospectively evaluated 86 patients with CRPC who received cabazitaxel at Showa Medical University–affiliated hospitals between 2015 and 2021. Cabazitaxel was administered every 3‐4 weeks at doses determined by the treating physicians, generally 20–25 mg/m^2^, with daily oral prednisolone. Pegfilgrastim was administered to all patients at least 24 h after cabazitaxel. Univariate analyses were performed to identify baseline factors associated with FN occurring during the first cycle of cabazitaxel. Exploratory logistic regression analysis was additionally performed for FN occurring during the entire treatment course.

**Results:**

Among the 86 patients, FN occurred in 12 patients (14.0%) during the entire treatment course, including 8 events (9.3%) during the first cycle. In univariate analyses, the only factor significantly associated with first‐cycle FN was a longer interval from CRPC diagnosis to cabazitaxel initiation (median, 1194.5 vs. 689 days, *p* = 0.0094). A similar tendency was observed when FN occurrence during the entire treatment course was analyzed. In exploratory logistic regression analysis for FN during the entire treatment course, the same factor showed a consistent trend toward association with FN development.

**Conclusion:**

Despite universal pegfilgrastim prophylaxis, approximately 10% of Japanese patients developed FN during the first cycle of cabazitaxel therapy. A longer interval from CRPC diagnosis to cabazitaxel initiation showed a consistent trend toward association with FN risk in Japanese patients across analyses. These findings provide real‐world safety data and highlight the importance of careful hematologic monitoring, particularly in patients treated later in the CRPC disease course.

## 1. Introduction

There are many drug treatment options for metastatic castration‐resistant prostate cancer (CRPC); however, the optimal order of administration in sequential therapy has not been clearly defined. A large‐scale Phase III clinical trial (TROPIC) [1] demonstrated that cabazitaxel significantly improved survival in patients with metastatic CRPC whose disease progressed after docetaxel treatment. However, this trial did not include Japanese institutions, in which only 7% of participants were Asian.

In a Japanese Phase I clinical trial (TED11576), hematologic toxicity of Grade 3 or higher was frequently observed, with 100% of patients developing neutropenia and 54.5% developing febrile neutropenia (FN) [2]. In that study, granulocyte colony–stimulating factor (G‐CSF) was not administered prophylactically during the first cycle. Considering that the overall incidence of FN was only 7.5% in the TROPIC trial, which predominantly enrolled Caucasian patients, previous Japanese studies have suggested a potentially higher risk of FN associated with cabazitaxel in Japanese patients.

Filgrastim, a short‐acting G‐CSF, was modified to create the long‐acting formulation pegfilgrastim. A Japanese postmarketing surveillance study also reported a relatively high incidence of FN despite increasing use of prophylactic G‐CSF, suggesting that Japanese patients might have greater susceptibility to cabazitaxel‐induced myelosuppression [3]. In addition, the prospective PEGAZUS study demonstrated that pegfilgrastim effectively reduced FN incidence in Japanese patients receiving cabazitaxel [4]. The American Society of Clinical Oncology recommends primary G‐CSF prophylaxis when the FN risk exceeds 20% [5]. Accordingly, pegfilgrastim is commonly used for primary prophylaxis in patients undergoing cabazitaxel therapy, and its administration is now considered almost essential.

In this study, we investigated the safety of cabazitaxel therapy with primary prophylactic pegfilgrastim in Japanese patients with CRPC, with a particular focus on the development of FN.

## 2. Materials and Methods

### 2.1. Study Design and Patients

This retrospective study included 86 patients who started cabazitaxel treatment for CRPC at Showa Medical University–affiliated facilities between January 2015 and December 2021 and were administered pegfilgrastim. The cabazitaxel dose was generally 20–25 mg/m^2^ every 3–4 weeks, and prednisolone (10 mg) was administered daily. The initial cabazitaxel dose was selected at the discretion of the treating physician based on the patient’s clinical condition. Pegfilgrastim was administered to all patients subcutaneously at least 24 h after cabazitaxel administration. The primary endpoint was the incidence of neutropenia of Grade 3 or higher and FN. The predictors of FN onset were investigated in patients with and without FN.

Clinical information, laboratory data, prior treatments, and treatment outcomes were collected from medical records.

### 2.2. Treatment Protocol

Cabazitaxel was administered every 3‐4 weeks at doses selected by the treating physician, generally between 20 and 25 mg/m^2^ combined with daily prednisolone (10 mg). Pegfilgrastim was subcutaneously administered at least 24 h after cabazitaxel administration in all patients. Dose reductions and delays were performed as clinically indicated.

### 2.3. Definition of FN

FN was defined as an axillary temperature ≥ 37.5°C accompanied by an absolute neutrophil count (ANC) < 500/μL, or an ANC < 1000/μL with an expected decline to < 500/μL within 48 h, according to established clinical practice guideline [5].

### 2.4. Endpoints

The primary endpoint was the occurrence of FN during the first cycle of cabazitaxel, because all baseline clinical variables were assessed at treatment initiation and early‐cycle FN was the most relevant to decisions about G‐CSF prophylaxis. The overall incidence and timing of FN during the entire treatment course were also described.

### 2.5. Statistical Analysis

Univariate analyses were performed to explore factors associated with first‐cycle FN. The following baseline factors were evaluated: age, performance status (PS), prostate‐specific antigen (PSA) at cabazitaxel initiation, grade group (International Society of Urological Pathology [ISUP]), baseline neutrophil count, hemoglobin level, serum albumin level, cumulative docetaxel dose before cabazitaxel, and the interval from CRPC diagnosis to cabazitaxel initiation. Categorical variables were analyzed using Fisher’s exact test, while continuous variables were compared using the Mann–Whitney *U* test. Because only eight FN events occurred during the first cycle, multivariable logistic regression for the primary endpoint was considered statistically unreliable and was not performed. Instead, exploratory logistic regression analysis was conducted for FN occurring during the entire treatment course to provide a regression‐based assessment with a larger number of events. Model calibration was assessed using the Hosmer–Lemeshow goodness‐of‐fit test. A two‐sided *p* value < 0.05 was considered statistically significant. The discriminative ability of the multivariable model was evaluated using the area under the receiver operating characteristic (ROC) curve (AUC).

Statistical analyses were performed using JMP Pro (Version 15, SAS Institute, Cary, NC, USA).

### 2.6. Ethics

This study was approved by the Institutional Review Board of Showa Medical University (No. 21‐170‐A). Informed consent was obtained through an opt‐out process in accordance with institutional policies and the principles of the Declaration of Helsinki.

All references were checked for retraction status and postpublication corrections using PubMed and the Retraction Watch DATABASE prior to submission. No cited articles were found to be retracted; moreover, no corrections that would have affected the relevance of the references in the context of this study were identified.

## 3. Results

The median time from CRPC diagnosis to cabazitaxel initiation was 798 days (range: 146–3942). Before cabazitaxel treatment, the patients had received a median of 6 docetaxel cycles (range: 1–77), with a median cumulative docetaxel dose of 600 mg/m^2^ (range: 75–8470). FN during prior docetaxel treatment occurred in 17 patients (19.8%). The median number of cabazitaxel cycles administered was 5 (range: 1–24). Overall, 12 patients (14.0%) developed FN at any time during the cabazitaxel treatment course. During the first cycle, 33 patients (41.5%) experienced Grade ≥ 3 neutropenia, and 8 patients (9.3%) developed FN. FN occurred in one patient each during the second, third, seventh, and ninth cycles.

Baseline patient characteristics of the entire cohort are summarized in Table 1. Comparisons between patients with and without FN during the entire treatment course are presented in Table 2. No significant differences were observed in age, PSA levels, grade group at diagnosis, visceral metastasis, laboratory parameters, or cumulative docetaxel dose between the FN and non‐FN groups.

**TABLE 1:** Baseline characteristics of patients according to febrile neutropenia during the first cycle of cabazitaxel.

	FN group (*n* = 8)	Non‐FN group (*n* = 78)	*p* value
Age at diagnosis, years, median	66 (60–76)	68 (44–84)	0.6624
PSA at diagnosis (ng/mL), median	68.95 (3.384–7039)	117.4 (2.880–10000)	0.9763
Grade Group 4‐5 (ISUP) at diagnosis, *n* (%)	4 (50.0)	61 (78.2)	0.1178
Visceral metastasis, *n* (%)	3 (37.5)	15 (19.2)	0.3555
Time from CRPC to CBZ initiation, days, median	1194.5 (877–1981)	689 (146–3942)	0.0094
Cumulative DTX dose (mg/m^2^), median	805 (280–1120)	593 (75–8470)	0.8219
Age at the time of CBZ administration, years, median	72 (63–86)	74 (46–89)	0.6338
PSA at the time of CBZ initiation (ng/mL), median	69.1 (2.4–504)	46.3 (0.2–4233)	0.2968
Initial CBZ dose:25 mg/m^2^, *n* (%)	2 (25)	17 (22)	1.000
Before CBZ administration			
Absolute neutrophil count (/µL), median	3144.5 (2038–4180)	4092 (1640–10662)	0.2044
Hemoglobin (g/dL), median	12.5 (8.4–13.1)	12.05 (6.3–15.3)	0.8003
Albumin (g/dL), median	4.0 (2.8–4.4)	3.7 (2.5–4.6)	0.5057

*Note:* CBZ, cabazitaxel; DTX, docetaxel.

Abbreviation: ECOG‐PS, Eastern Cooperative Oncology Group performance status.

**TABLE 2:** Exploratory multivariable logistic regression analysis of factors associated with febrile neutropenia during the entire treatment course.

	OR	95% CI	*p* value
Age at CBZ initiation (years)	0.97	0.89–1.05	0.435
Time from CRPC to CBZ initiation (days)	1.00	1.00–1.00	0.062

**FIGURE 1:**
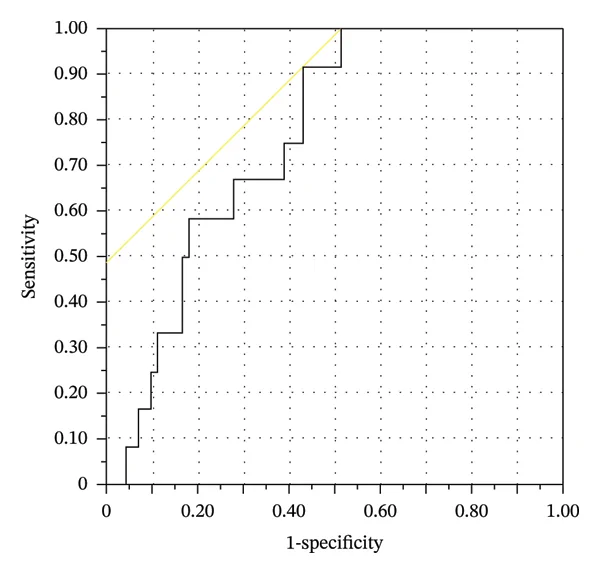
Receiver operating characteristic curve of the multivariable logistic regression model for febrile neutropenia during the entire treatment course. Receiver operating characteristic (ROC) curve of the multivariable logistic regression model for febrile neutropenia during the entire treatment course. The model included age at cabazitaxel initiation and the interval from CRPC diagnosis to cabazitaxel initiation. The yellow line represents the optimal cut point (Youden’s J) automatically generated by the software, indicating the threshold that maximizes the sum of sensitivity and specificity. It does not represent the diagonal chance reference line. The area under the curve (AUC) was 0.76.

Patient characteristics stratified by first‐cycle FN are presented in Table 3. In univariate analyses, age, PS, grade group at diagnosis, baseline laboratory parameters (absolute neutrophil count, hemoglobin, and serum albumin), cumulative docetaxel dose, and initial cabazitaxel dose were not significantly associated with the occurrence of FN during the first cycle of cabazitaxel treatment. The only factor significantly associated with first‐cycle FN was a longer interval from CRPC diagnosis to cabazitaxel initiation (median, 1194.5 vs. 689 days, *p* = 0.0094). When FN occurring during the entire treatment course was analyzed using exploratory logistic regression, a longer interval from CRPC diagnosis to cabazitaxel initiation showed a consistent trend toward association with FN development, although statistical significance was not reached. Comparisons between patients with and without FN during the entire treatment course are presented in Table 2. No other baseline clinical or treatment‐related variables were associated with FN development. The Hosmer–Lemeshow goodness‐of‐fit test indicated an adequate model fit (*p* = 0.904). The discriminative ability of the multivariable model was moderate, with an AUC of 0.76 (Figure 1).

**TABLE 3:** Univariate logistic regression analysis of factors associated with febrile neutropenia during the first cycle of cabazitaxel.

	OR	95% CI	*p* value
Age at CBZ initiation (years)	0.97	0.90–1.05	0.454
PSA at CBZ initiation (ng/mL)	1.00	1.00–1.00	0.707
Grade Group (ISUP)	2.07	0.40–8.70	0.355
Time from CRPC to CBZ initiation (days)	1.00	1.00–1.00	0.066
Cumulative DTX dose (mg/m^2^)	1.00	1.00–1.00	0.964
CBZ dose administered (25 mg/m^2^ vs < 25 mg/m^2^)	1.20	0.17–5.75	0.837
Before CBZ administration			
Absolute neutrophil count (/µL)	1.00	1.00–1.00	0.216
Hemoglobin (g/dL)	1.06	0.76–1.54	0.737
Albumin (g/dL)	1.72	0.28–14.0	0.570

Abbreviations: CI, confidence interval; OR, odds ratio.

## 4. Discussion

Among currently available treatments that have demonstrated survival benefits in patients with metastatic CRPC—including abiraterone/prednisolone [6], enzalutamide [7], docetaxel [8], cabazitaxel [1, 9], radium‐223 [10], olaparib [11], and pembrolizumab [12]—the optimal sequencing strategy in routine clinical practice has not been clearly established.

Cabazitaxel is a next‐generation taxane that has demonstrated efficacy in docetaxel‐resistant disease cases. In the pivotal Phase III TROPIC trial, cabazitaxel significantly improved overall survival compared to mitoxantrone in patients with docetaxel‐resistant metastatic CRPC [1]. However, this trial did not include Japanese institutions; only a small proportion of participants were Asian. Subsequent Japanese studies have suggested that hematologic toxicity, particularly FN, might occur more frequently in Japanese patients receiving taxane‐based chemotherapy [2].

FN incidence in Japanese patients treated with docetaxel has been reported to be higher than that observed in large Western Phase III trials [13], whereas Phase II studies conducted in Japan have shown FN rates as high as 16.3% [14]. These findings suggest potential ethnic differences in susceptibility to taxane‐induced myelosuppression.

Although a Phase III randomized trial demonstrated that cabazitaxel was not superior to docetaxel in taxane‐naïve patients [15], cabazitaxel remains an important treatment option for managing patients with disease progression after docetaxel. Furthermore, the Phase III PROSELICA trial demonstrated that a reduced dose of cabazitaxel (20 mg/m^2^) was noninferior to the standard dose (25 mg/m^2^) in terms of overall survival, supporting the clinical feasibility of dose adjustment according to patient condition [16].

To mitigate hematologic toxicity, prophylactic administration of G‐CSF has been recommended during cabazitaxel therapy [17]. In Japan, prospective and postmarketing studies have demonstrated that pegfilgrastim prophylaxis substantially reduces the incidence of FN [3, 4]. In the present study, FN occurred in 14.0% of patients during the entire treatment course and in 9.3% during the first cycle, despite universal pegfilgrastim prophylaxis. These results are consistent with previously reported Japanese real‐world data [4] and confirm that FN remains a clinically relevant adverse event in this population. The most notable finding of this study was that a longer interval from CRPC diagnosis to cabazitaxel initiation showed a consistent trend toward association with FN development across analyses. This association was observed both for FN occurring during the first cycle and for FN occurring at any time during the treatment course. In contrast, other baseline clinical factors and treatment‐related variables—including age, laboratory parameters, cumulative docetaxel dose, and initial cabazitaxel dose—were not significantly associated with FN.

The relationship between a longer CRPC disease course and FN development may reflect cumulative treatment exposure, gradual deterioration in bone marrow reserve, or underlying disease biology not fully captured by baseline laboratory parameters. In this context, a previous Japanese retrospective study reported that sarcopenia and visceral metastasis at the time of cabazitaxel initiation were independent predictors of poor prognosis, suggesting that delayed introduction of cabazitaxel may reflect advanced disease status and impaired physiological reserve [18]. Although that study focused on survival outcomes rather than treatment‐related toxicity, its findings support the concept that patients with later‐stage CRPC may be more vulnerable to adverse events during cabazitaxel therapy. Notably, baseline neutrophil count and cumulative docetaxel dose were not associated with FN, suggesting that FN risk during cabazitaxel therapy cannot be explained solely by measurable hematologic indices or prior taxane exposure.

In the exploratory multivariable analysis, the interval from CRPC diagnosis to cabazitaxel initiation showed a consistent trend toward association with FN development. The model demonstrated acceptable calibration, as indicated by the Hosmer–Lemeshow goodness‐of‐fit test, and moderate discriminative ability with an area under the ROC curve (AUC) of 0.76. These findings support the robustness of the observed association, although the results should be interpreted cautiously given the limited number of events.

Previous studies have discussed the balance between toxicity and relative dose intensity (RDI) of cabazitaxel. Terada et al. reported that higher initial cabazitaxel doses (≥ 22.5 mg/m^2^) were associated not only with increased Grade 4 neutropenia but also with higher RDI and longer overall survival in Japanese patients [19]. Similarly, Kosaka et al. reported that the absence of Grade 3/4 neutropenia during cabazitaxel treatment may indicate insufficient drug exposure [20]. However, this study was not designed to assess the impact of cabazitaxel dose or dose intensity on FN risk or treatment outcomes.

Several limitations should be acknowledged. This study was retrospective in nature and subject to selection bias. The cabazitaxel dose was determined by the treating physician, and treatment efficacy outcomes were not evaluated in relation to FN development. In addition, the limited number of FN events precluded reliable multivariable analysis. Furthermore, all patients received pegfilgrastim prophylaxis; therefore, the direct impact of pegfilgrastim could not be assessed. Despite these limitations, this study represents one of the largest real‐world cohorts evaluating the safety of cabazitaxel with universal pegfilgrastim prophylaxis in Japanese patients with CRPC. In addition, FN was analyzed as a binary outcome in this study, and the timing of FN onset was not formally evaluated using time‐to‐event methods; future studies incorporating time‐dependent analyses may provide a more comprehensive understanding of FN risk during cabazitaxel treatment. In conclusion, FN remains a clinically relevant adverse event in Japanese patients treated with cabazitaxel, even when primary pegfilgrastim prophylaxis is routinely administered. A longer interval from CRPC diagnosis to cabazitaxel initiation showed a trend toward increased FN risk, underscoring the importance of careful hematologic monitoring, particularly in patients treated later in the CRPC disease course.

## Author Contributions

Kazuhiko Oshinomi: methodology, software, data curation, writing–original draft, writing–review and editing, and visualization. Shota Kikuchi, Masahiro Kurokawa, Toshiki Mugita, Tatsuki Inoue, and Motoki Yamagishi: data curation. Yoshihiro Nakagami: data curation, supervision, and investigation. Masakazu Nagata, Haruaki Sasaki, and Kohzou Fuji: supervision. Masashi Morita: data curation and supervision. Takashi Fukagai: supervision and project administration.

## Funding

No funding was received for this manuscript.

## Disclosure

All authors have read and approved the final version of the manuscript. The corresponding author had full access to all of the data in this study and takes complete responsibility for the integrity of the data and the accuracy of the data analysis. No funding bodies or commercial entities had any role in the study design; collection, analysis, or interpretation of data; writing of the manuscript; or the decision to submit the manuscript for publication.

## Ethics Statement

The corresponding author affirms that this manuscript is an honest, accurate, and transparent account of the study being reported; that no important aspects of the study have been omitted; and that any discrepancies from the study as planned have been explained.

This study was approved by the Institutional Review Board of Showa Medical University (no. 21‐170‐A).

## Consent

Informed consent was obtained by an opt‐out approach.

## Conflicts of Interest

The authors declare no conflicts of interest.

## Data Availability

The data supporting the findings of this study contain sensitive patient information and are therefore not publicly available. Data may be made available from the corresponding author upon reasonable request and with appropriate institutional approval.
